# DenseMatch: a dataset for real-time 3D reconstruction

**DOI:** 10.1016/j.dib.2021.107476

**Published:** 2021-10-14

**Authors:** Marco Lombardi, Mattia Savardi, Alberto Signoroni

**Affiliations:** Information Engineering Department, University of Brescia – Via Branze 38, Brescia 25123, Italy

**Keywords:** 3D registration, Point-based, Real-time 3D reconstruction, Benchmark dataset

## Abstract

We provide a database aimed at real-time quantitative analysis of 3D reconstruction and alignment methods, containing 3140 point clouds from 10 subjects/objects. These scenes are acquired with a high-resolution 3D scanner. It contains depth maps that produce point clouds with more than 500k points on average. This dataset is useful to develop new models and alignment strategies to automatically reconstruct 3D scenes from data acquired with optical scanners or benchmarking purposes.

**Specifications Table**

**Table:** 

Subject	Computer Graphics and Computer-Aided Design, Computer Vision and Pattern Recognition
Specific subject area	Small scale object reconstruction from dense 3D data
Type of data	Binary file (.npz format) containing Pointclouds (.ply format) and metadata Depth map images (color images in.jpg format and depth images in.png format)
How data were acquired	All the data were acquired with a high-quality handheld 3D optical scanner
Data format	Raw data Converted to Depth map
Parameters for data collection	The dataset consists of small-scale acquisitions of objects including human subject portions. The acquisitions include the raw flow of depth data coming from the device, with natural interruptions and a non-constant acquisition speed, in accordance to what happens in real use cases
Description of data collection	The database contains 3140 point clouds composing 19 scenes, taken by scanning 10 different subjects acquired in a controlled environment. The data has a resolution up to 1280×1024 and depth resolution up to 1 mm. Each depth map produces 500k points on average, with a field of view of the scanner around 300×300 mm
Data source location	Institution: University of Brescia City/Town/Region: Brescia Country: Italy Latitude and longitude for collected samples/data: 45.56466; 10.231660
Data accessibility	Repository name: Data in Brief Harvard Dataverse Data identification number: CU4UXG Direct URL to the data: 10.7910/DVN/CU4UXG Direct URL to the code: 10.5281/zenodo.5534851
Related research article	M. Lombardi, M. Savardi, A. Signoroni, *Cross-domain assessment of deep learning-based alignment solutions for real-time 3D reconstruction*, Computers & Graphics (2021) https://doi.org/10.1016/j.cag.2021.06.011

## Value of the Data

• The data are useful to develop models and alignment strategies to automatically reconstruct 3D scenes from data acquired with optical scanners.
• The data can be used by computer scientists to perform quantitative analysis of 3D reconstruction and alignment methods.
• By using these data it is possible to train or fine-tune AI models to cope with small-scale and dense 3D object acquisitions and reconstructions.
• The dimension of this dataset is considerable, being composed of 19 scenes made on 10 different subjects. It contains depth maps that produce point clouds with more than 500k points on average.

## 1 Data Description

3D reconstruction aims at retrieving the geometric context and description of a scene inspected by a scanner. A lot of reasons motivate such goal, *e.g.* object modelling, reverse engineering, industrial quality inspection, cultural heritage, building information modelling, odometry for robot movement, virtual reality, etc. According to the task and to the domain requirements, there exists a large variety of scanners to address each of those applications. A very interesting solution is given by so-called handheld 3D scanners, which are portable devices that can be moved around the scene to acquire and model 3D targets, providing feedback to the user in a real-time fashion. These instruments are typically lightweight, they work at a relatively high frame rate and they are meant to target problems such as product design, reverse engineering, personalised orthopaedics, cultural heritage documentation, to name a few. Moreover, they are differentiated according to the type of sensor in use. A very common sensing device is the optical 3D scanner, which usually employs one or multiple cameras to infer a 2D representation of the scene which is later mapped into 3D. The accuracy and the quality of reconstruction for this kind of device have grown together with the incremental availability of higher computational power at a commodity price and this has raised the interest around them. Modern handheld optical scanners are able to provide high-detailed scans, which allows for much more accurate reconstructions than was possible in the recent past.

On top of the growing interest in this kind of technology, researchers have spent a lot of efforts addressing some of the more challenging tasks related to on-the-fly 3D reconstructions, such as the problem of view alignment (or view registration). In more recent years, the advent of deep learning has invested many research communities, including the one of Computer Vision. In particular, only very recently 3D data analysis has been successfully addressed by data-driven approaches. However, to the best of our knowledge, the scenarios which have been dealt with this kind of solutions are typically related to the problems of indoor or outdoor reconstruction (i.e. at medium to large scales). Therefore, popular reference datasets are composed of relatively large scenes. Moreover, such datasets usually derive either from commodity handheld optical scanners, with limited camera resolution, or from laser scanners with a large sensing range and sparse data distribution. Differently, main interests in the case of small-scale 3D object/subject models are usually focused on themes related to semantic segmentation, detection, and classification. In such scenarios, the 3D data are often acquired via photogrammetric pipelines or synthetically generated with CAD tools.

Starting from these considerations, we decided to propose a collection of small-scale scenes acquired with a handheld 3D optical scanner that provides dense and more accurate data, in order to provide good fresh data to the community interested in investigating dense 3D object reconstruction algorithms.

**Fig. 1:**
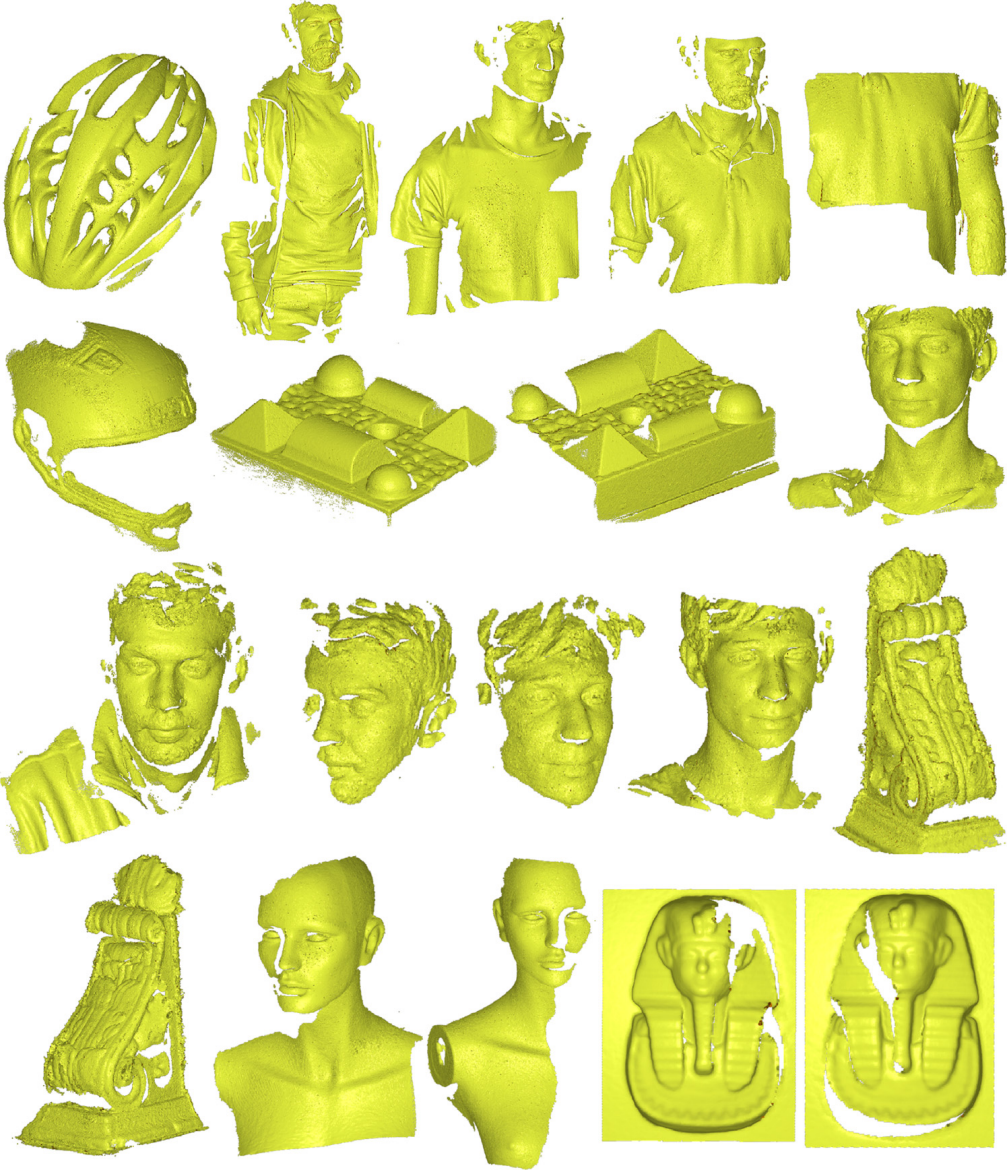
Set of scenes contained in DenseMatch. From top left corner to bottom right: bike helmet, body 1, body 4, body 2, body 3, helmet, geom 1, geom 2, face 5, face 1, face 2, face 3, face 4, column 1, column 2, dummy 1, dummy 2, statue 1 and statue 2.

The raw data streamed during the scan session are remarkably dense: each depth map produces 500k points on average, all bounded in a small box, since the field of view of the scanner is around 300×300 mm. In Table 1 we report additional information for a schematic overview. The volume of the bounding box gives a hint about the size of each scan. The number of points per frame, together with the spacing of the points, provides a hint of the point cloud density. The latter is evaluated by computing the average distance between the points in a point cloud. All of these elements are reported with the minimum, maximum, and average values for the whole dataset. As reported in the table 1 we collected a considerable set of 3140 point clouds, divided into 19 scenes, which have been taken by scanning 10 different subjects. Those subjects comprise human bodies and faces, reproduction of artworks, and design objects. Each scene of DenseMatch is shown in Fig. 1.

**Table 1:** Main specifications of the dataset. Inside the parenthesis the average values, while in the square brackets the ranges.

		notes

Scanner Type	Handheld Optical Scanner	Stereo infra-red Cameras (resolution 1280×1024) + RGB Cam
# Point Clouds	3140	collected on 19 scenes, targeting object reconstruction
# Points per PC	[60k - 1M] (500k)	[range from minimum to maximum value] (average on all dataset)
BBox Volume per PC	[0.0001 - 0.01] (0.003) m${}^{3}$	Oriented Bounding Box estimated using Open3D [3]
Point Spacing per PC	[0.00018 - 0.00035] (0.00025) m	Estimated with Open3D by averaging closest points-distances in each PC

The dataset [1] contains two type of files:

• *npz* files are Python readable files, stored in binary format using the Numpy open source package. Each file contains all the point clouds (in standard *ply* format) and additional metadata (name and pose) belonging to a specific scene. The total size of the folder containing the files is 18.4Gb.
• *tar.gz* is a large archive file (1.8 Gb) which contains the different folders that are labeled similarly to the point clouds. The archive was compressed using gzip algorithm, therefore it can be unzipped with any standard decompression tool. Each folder contains the RGB-D images and the camera calibration parameters that were used to reconstruct the 3D data. These images are the original output produced by the 3D scanner that was used to generate the data. In particular, the color images have *.jpg* extension, while the depth images are 16 bit *.png* files.

In the dataset repository we also provided a link to an open source code [2] for custom reader we implemented in Python. The reader works as a command line application which allows to specify different settings. For instance the user can choose whether to read a point cloud directly or to re-create it from scratch using the RGB-D images of a specific scene. Specifically, each RGB-D is wrapped into a Frame structure that contains color and depth images, camera pose and point cloud members. Each frame can also be saved into a Sequence class which contains the camera parameters and additional metadata. Such organized structure can be easily integrated with any research code the user intends to develop. Finally, a simple example is given to load a sequence and to visualize it. Everything is based on few very popular Python packages and the code should run effortless on classical environments, such as Windows, Linux and MacOS.

## 2 Experimental Design, Materials and Methods

**Fig. 2:**
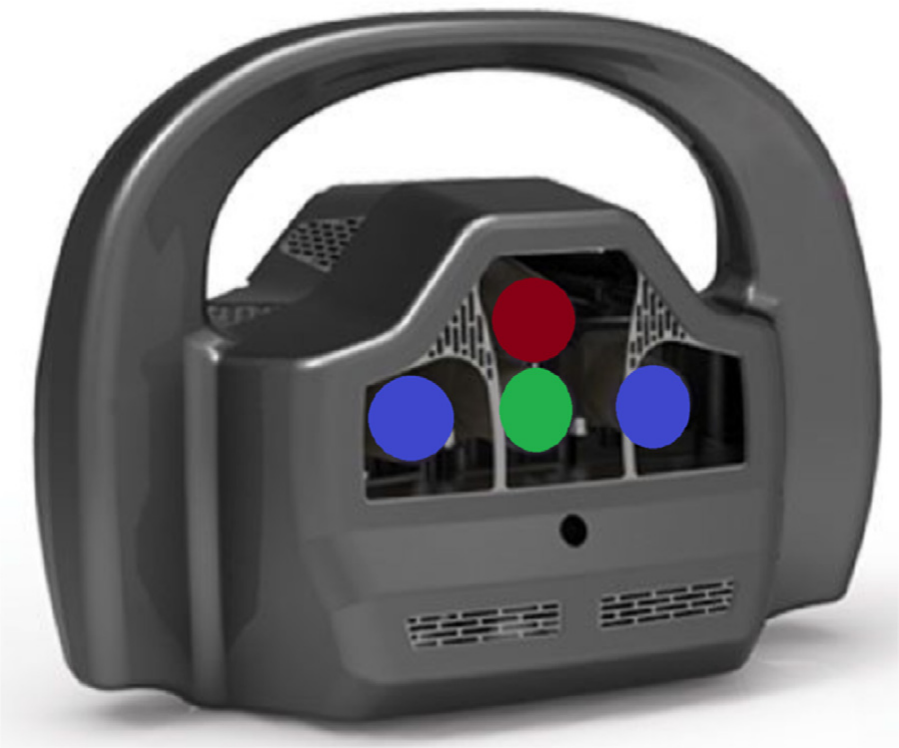
Render of the InSight3 scanner prototype. In blue are highlighted the two infra-red cameras, in red the infra-red projector and in green the RGB camera.

To create the dataset, we used a non-commercial prototype *InSight3* (see Fig. 2), an optical scanner able to acquire 3D shapes via stereo triangulation by means of a pair of infra-red cameras with resolution up to 1280×1024 and depth resolution up to 1 mm. The scanner is connected to a computer via USB 2.0 and Ethernet connectors. The synchronisation of the cameras is made on-board, along with the start and stop buttons triggering. On the contrary, the image processing and the 3D modelling are performed on the connected PC, leveraging GPU computation. Our setup comprises a machine running Windows 10 with 16GB of RAM and an Nvidia GTX 1080 Ti.

**Fig. 3:**
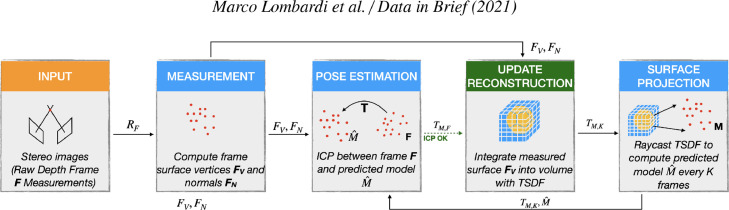
Schematic acquisition workflow.

**Fig. 4:**
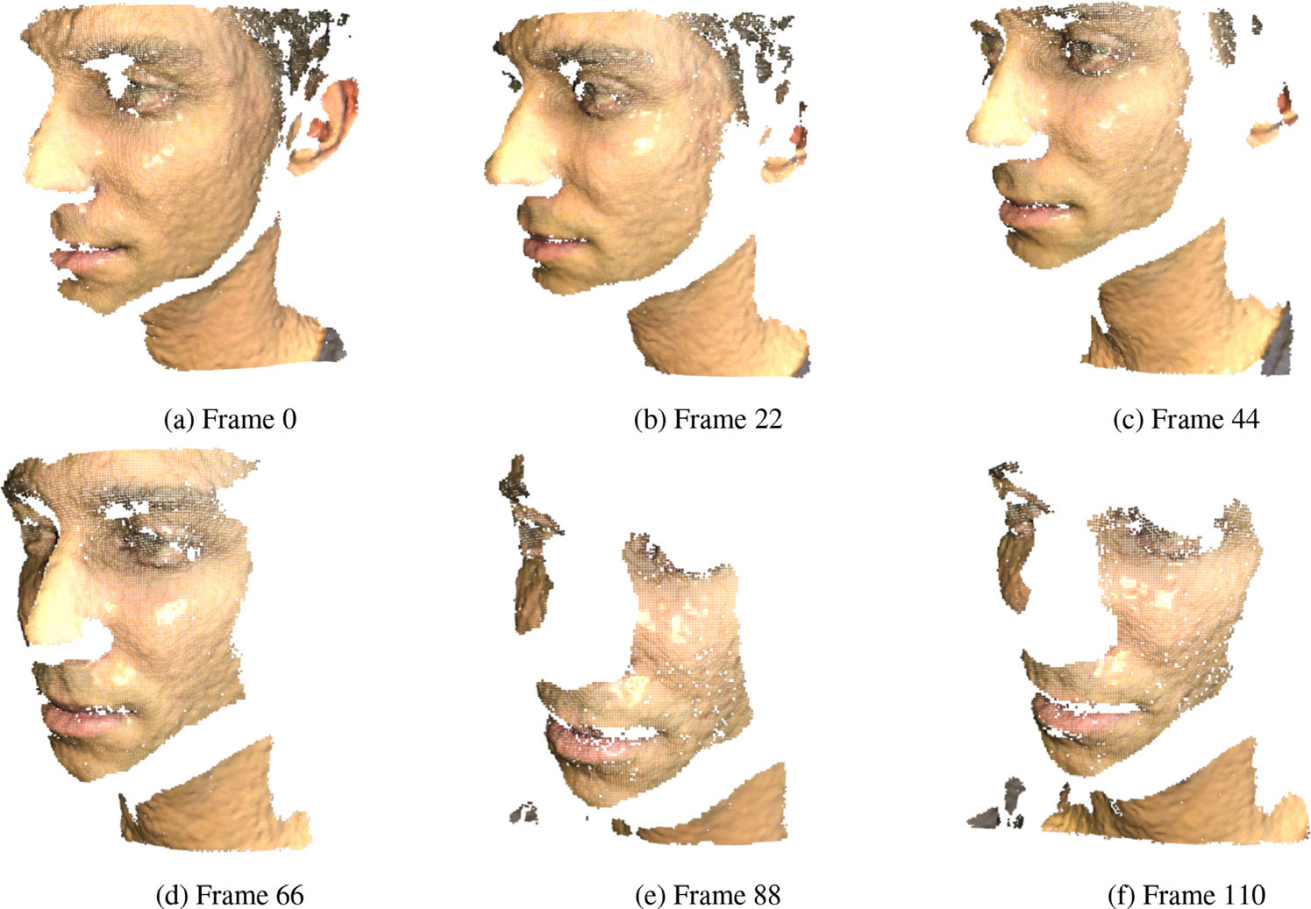
Subset of frames acquired during the scan session on face 5 scene (114 frames total).

A schematic representation of the workflow is given in Fig. 3. The pipeline is similar to what was presented in KinectFusion [4] and its further improvement based on voxel hashing for scaling 3D data structure. It starts from the acquisition of a pair of stereo images via infra-red cameras. During the acquisition, a pseudo-random pattern is projected by means of an infra-red projector in order to add a synthetic texture to the scene. This is known as an *active* approach to the 3D scanning and it is helpful to disambiguate the point matching stage required during the triangulation process. The calibrated setup leverages the epipolar geometry constraints to produce a disparity map which is then used to create a surface map via stereo triangulation. Such a map stores pixel-wise 3D coordinate information, having the same size of the camera sensor. Surface normal information is estimated for each point and added thanks to the an efficient Principal Component Analysis implementation on the covariance matrix built upon the nearest neighbours of the query point. Eventually, the supplementary RGB camera allows for adding texture to the data. Both intrinsic and color calibration are performed beforehand by means of the calibration software provided with the scanner. An example of a small set of frames belonging to a scene of the dataset is given in Fig. 4.

A volumetric approach, inspired by the work of Curless and Levoy [5], is used to integrate the acquired 3D views and to update the model in a real-time fashion. In order to properly integrate the data, it is necessary to retrieve a fine estimation of the pose of the camera in the world, since the sensed 3D data is originally referred to as the camera coordinate system. To do this, it is possible to assume that the current frame is already close to the already acquired model, because of the short time elapsing between two consecutive 3D frame acquisitions. Therefore, a fine local registration method based on ICP [6] is used to register the new frame. The registration method is called frame-to-model since the current frame is registered against the latest 3D projection of the model via ray-casting, as suggested in [4]. The ray-casting is also used to give a real-time feedback to the user which is able to look at the growing model on a screen during the acquisition.

The system nominally acquires 10 frames per second. As already mentioned, during the process every pair of stereo images is triangulated to produce a range image containing color and depth information. Due to the necessity of saving each range image on disk for further post-processing, the frame rate decreased a bit overall. After each scanning session, we ended up with a collection of range images stored in a proprietary format which we then split into color and depth images. These images can be further passed to open source libraries such as Open3D [3] for different purposes. In post-processing we proceeded as follows: first, we extracted a point cloud for each frame. Then we performed a direct alignment for all the point clouds composing the scene, by following the implementation of [7], which is a feature-based pipeline the leverages ICP [6] for 3D alignment. Then we fixed by hand the failed alignments that occurred during the process by running again the pipeline with user-selected control points for the critical point clouds. Finally, we refined the result in a global fashion by leveraging on an optimization-on-a-manifold framework [8]. Eventually, the final set of point clouds is properly aligned, thus constituting the ground truth for different kinds of tests on 3D object registration or for training/fine-tuning models to cope with small-scale 3D objects and dense acquisitions.

We already used DenseMatch in two studies [9], [10] focused on DL-based 3D alignment and real-time 3D reconstruction. In these works, the point clouds were initially misaligned by means of a supervised random perturbation of the ground truth poses. In particular, the random rotations spanned the whole range of angles from 0 to 2π onto the 3 axes. Additionally, a random translation was also produced, again on the 3 axes within a range of ±1 m. The misaligned data served as input to 3D reconstruction pipelines addressing the problem of 3D registration where we performed a cross-domain assessment of deep learning-based solutions. We point out that the set of transformations applied in the above-referenced works is also available as metadata in the provided dataset.

We encourage the research community to exploit DenseMatch. In the meantime, we are willing to increase the pool of scenes in the future.

## Ethics Statement

Informed consent was obtained for the scenes in which a human subject is present. The portrayed individual gave consent in publishing Fig. 4.

## CRediT authorship contribution statement

**Marco Lombardi:** . **Mattia Savardi:** . **Alberto Signoroni:** Conceptualization, Visualization, Supervision, Writing – review & editing, Project administration.

## Declaration of Competing Interest

The authors declare that they have no competing financial interests or personal relationships which have, or could be perceived to have, influenced the work reported in this article.
